# Retention in Individual Trauma-Focused Treatment Following Family-Based Treatment Among US Veterans

**DOI:** 10.1001/jamanetworkopen.2023.49098

**Published:** 2023-12-21

**Authors:** Jessica Dodge, Kathrine Sullivan, Peter P. Grau, Charity Chen, Rebecca Sripada, Paul N. Pfeiffer

**Affiliations:** 1 Health Services Research and Development/Center for Clinical Management Research, Ann Arbor Veterans Affairs Hospital (152), Ann Arbor, Michigan; 2 Silver School of Social Work, New York University, New York; 3 Department of Psychiatry, University of Michigan Medical School, Ann Arbor

## Abstract

**Importance:**

Despite the availability of several empirically supported trauma-focused interventions, retention in posttraumatic stress disorder (PTSD) psychotherapy is poor. Preliminary efficacy data shows that brief, family-based interventions may improve treatment retention in a veteran’s individual PTSD treatment, although whether this occurs in routine clinical practice is not established.

**Objective:**

To characterize receipt of family therapy among veterans diagnosed with PTSD and evaluate whether participation in family therapy is associated with an increased likelihood of completing individual trauma-focused treatment.

**Design, Setting, and Participants:**

This retrospective cohort study used the Veterans Health Administration (VHA) Informatics and Computing Infrastructure to extract electronic health record data of participants. All participants were US veterans diagnosed with PTSD between October 1, 2015, and December 31, 2019, who attended at least 1 individual trauma-focused treatment session. Statistical analysis was performed from May to August 2023.

**Exposures:**

Receipt of any family psychotherapy and subtype of family-based psychotherapy.

**Main Outcomes and Measures:**

Minimally adequate individual trauma-focused treatment completion (ie, 8 or more sessions of trauma-focused treatment in a 6-month period).

**Results:**

Among a total of 1 516 887 US veterans with VHA patient data included in the study, 58 653 (3.9%) received any family therapy; 334 645 (23.5%) were Black, 1 006 168 (70.5%) were White, and 86 176 (6.0%) were other race; 1 322 592 (87.2%) were male; 1 201 902 (79.9%) lived in urban areas; and the mean (SD) age at first individual psychotherapy appointment was 52.7 (15.9) years. Among the 58 653 veterans (3.9%) who received any family therapy, 36 913 (62.9%) received undefined family therapy only, 15 528 (26.5%) received trauma-informed cognitive-behavioral conjoint therapy (CBCT) only, 5210 (8.9%) received integrative behavioral couples therapy (IBCT) only, and 282 (0.5%) received behavioral family therapy (BFT) only. Compared with receiving no family therapy, the odds of completing individual PTSD treatment were 7% higher for veterans who also received CBCT (OR, 1.07 [95% CI, 1.01-1.13]) and 68% higher for veterans received undefined family therapy (OR, 1.68 [95% CI, 1.63-1.74]). However, compared with receiving no family therapy care, veterans had 26% lower odds of completing individual PTSD treatment if they were also receiving IBCT (OR, 0.74 [95% CI, 0.66-0.82]).

**Conclusions and Relevance:**

In this cohort study of US veterans, family-based psychotherapies were found to differ substantially in their associations with individual PTSD psychotherapy retention. These findings highlight potential benefits of concurrently providing family-based therapy with individual PTSD treatment but also the need for careful clinical attention to the balance between family-based therapies and individual PTSD treatment.

## Key Points

**Question:**

Is participation in family therapy associated with completion of individual therapy among US veterans with posttraumatic stress disorder (PTSD)?

**Findings:**

In this national cohort study of 1 516 887 US veterans diagnosed with PTSD, approximately 4% received any family therapy. Among those who attended at least 1 individual trauma therapy session, those who received trauma-informed or undefined family therapy during their individual trauma-focused treatment were associated with higher odds of completing a minimally adequate dose of individual treatment.

**Meaning:**

These results suggest that integration of trauma-informed family therapy into care for PTSD could help with retention in individual trauma-focused treatment.

## Teaser

This cohort study evaluates whether participation in family therapy is associated with an increased likelihood of completing individual trauma-focused treatment among US veterans with posttraumatic stress disorder.

## Introduction

The Veterans Health Administration (VHA) offers several evidence-based individual trauma-focused treatment options, including prolonged exposure therapy (PE) and cognitive processing therapy (CPT). Both treatments are effective at treating posttraumatic stress disorder (PTSD) in veterans.^1,2^ However, retention in trauma-focused treatments is a persistent problem (see Edwards-Stewart et al^3^ for systematic review and meta-analysis). Spouses and partners of veterans diagnosed with PTSD can strongly influence mental health treatment initiation,^4,5,6^ support treatment retention,^7^ and may help with treatment motivation.^8^ A lack of social support strongly contributes to the development and maintenance of PTSD.^9^ Therefore, engaging a veteran’s immediate social connections could be effective. For example, a recent study^6^ found that when family members of veterans with PTSD encouraged them to face difficult situations (a key component of trauma-focused treatment), veterans were twice as likely to finish PE or CPT.

Family relationships appear to have a particularly important role among active-duty military service members and veterans who experience greater disruptions in relational functioning due to PTSD symptoms compared with their nonmilitary counterparts. A meta-analysis of 31 studies investigating the associations between indicators of PTSD and intimate relationship problems found that the association between PTSD and relational discord was higher in military samples compared with civilian samples.^10^ There is strong evidence from systematic reviews documenting the effectiveness of couples therapies for treating adult PTSD and improving interpersonal relationship problems secondary to PTSD symptoms.^11,12^ However, these reviews were not specific to military or veteran populations. Considering evidence that associations between PTSD symptoms and relationship difficulties are stronger among military couples, and the potential for familial involvement to support veterans’ individual trauma-focused treatment, there is a need to understand how veterans are utilizing family therapy and the effect of family therapy on individual PTSD treatment. Several different family therapies are provided within the VHA; however, whether family treatment modalities differ regarding their potential association with individual PTSD treatment retention is unknown.

The VHA currently offers 3 empirically supported family therapies: (1) integrative behavioral couples therapy (IBCT), (2) behavioral family therapy (BFT), and (3) cognitive-behavioral conjoint therapy (CBCT).^13,14,15^ IBCT is 11 to 26 sessions, including a 4-session assessment phase before the start of treatment, and has a focus on acceptance between the couple and their relationship behaviors.^16,17^ BFT is 20 to 25 sessions and focuses on how an individual’s behavior is learned and reinforced in their environment; treatment focuses on changing behavior by changing the environmental feedback.^14,17^ Lastly, CBCT is 15 sessions and is designed to treat PTSD in a couples setting using principles of cognitive behavioral therapy applied to the couple.^17,18,19^ In order to understand the utilization of these therapies and test whether participation in each was associated with better outcomes in PTSD treatment, the current study used VHA electronic health record (EHR) data to address 2 research questions. (1) What family therapies are veterans diagnosed with PTSD using? (2) Is participation in family treatment associated with an increased likelihood of completing individual treatment among veterans with PTSD?

## Methods

### Participants and Procedure

The Veterans Affairs Ann Arbor Healthcare System institutional review board approved this cohort study and waived informed consent to access veterans’ protected health information via the VHA EHR. Informed consent was waived because it would not have been feasible to obtain consent from a very large, retrospective sample of patients. This study followed the Strengthening the Reporting of Observational Studies in Epidemiology (STROBE) reporting guideline.^20^

The VHA Informatics and Computing Infrastructure was used to extract EHR data from the corporate data warehouse (CDW) for all participants in the cohort. In addition to patient demographic information, the CDW contains *Current Procedural Terminology* and diagnosis codes for outpatient encounters and inpatient stays. The cohort for this study was defined as VHA patients diagnosed with PTSD between October 1, 2015, and December 31, 2019. *International Statistical Classification of Diseases and Related Health Problems, Tenth Revision (ICD-10)* diagnosis codes were used to identify patients who were diagnosed with PTSD in either an inpatient or outpatient setting during this period (see eTable 1 in Supplement 1 for a list of relevant *ICD-10* codes).

### Measures

The primary outcome was completion of a minimally adequate course of evidence-based psychotherapy (EBP) for PTSD (attended at least 8 sessions within a 6-month period). Although *Current Procedural Terminology* codes identify psychotherapy encounters, they are insufficient for determining specific treatment types. To support standardized delivery and tracking of EBPs in the VHA, clinicians are encouraged to use specific medical record note templates that identify the type of EBP within the health factors data field of the CDW. We queried this field to identify delivery of either PE or CPT, the 2 EBPs for PTSD with standardized note templates in the VHA. Patients who received at least 8 individual psychotherapy encounters for PE or CPT within any 6-month period were considered to have received a minimally adequate course of EBP for PTSD.

The primary independent variable was receipt of couples or family-based psychotherapy as determined by *Current Procedural Terminology* codes during the PE or CPT (see eTable 2 in Supplement 1 for a list of relevant *Current Procedural Terminology* codes). Evidence-based couples or family-based psychotherapy could consist of IBCT, BFT, or CBCT. We queried the health factors field for specific terms aligned with the national standardized note templates for IBCT and BFT (see eTable 3 in Supplement 1 for the list of family-based search terms used). However, there is no standardized note template for CBCT therapy for PTSD. To capture these visits, we searched for terms in the note contents (eg, “CBCT” or “Cognitive Behavioral Conjoint Therapy”) and excluded notes that contained the CBCT acronym but were not related to couples therapy (eg, excluded terms such as “dental” or “radiation”). Following an iterative process of refining these terms, we achieved 100% accuracy in pulling CBCT notes in a sample of 200 manually reviewed notes. Any veteran who had a *Current Procedural Terminology* code for couples or family therapy but was not included in our search for BFT, IBCT, or CBCT therapy was categorized as unspecified family therapy.

Demographic characteristics potentially associated with completing a minimally adequate course of individual psychotherapy treatment included age (continuous), sex (male vs female), and race (Black vs White vs other [American Indian or Alaska Native, Asian, Pacific Islander, other]); these variables were obtained from the EHR. Participants’ zip codes were also extracted and then merged with Rural-Urban Commuting Area code data from the US Department of Agriculture so that we could determine whether they lived in an urban or rural area (rural [codes 7-10], suburban [codes 4-6], urban [codes 1-3]). Military service history (including combat exposure and history of military sexual trauma) and service-connected disability (dichotomized into <50% vs ≥50% as a total disability rating that is on deciles, and it is not a continuous rating^21^) were also extracted from the EHR. Our model also assessed for comorbid psychiatric diagnoses, including depression, anxiety, substance use disorder, schizophrenia, bipolar disorder, and psychosis. All comorbid conditions were identified in the EHR using *ICD-10* codes. Lastly, we controlled receipt of other non-EBP psychotherapy visits (ie, case management) as an indicator of general mental health service utilization (see eTable 2 in Supplement 1 for *Current Procedural Terminology* codes used).

### Statistical Analysis

Descriptive statistics were calculated for all variables in the data set; summary statistics (eg, mean [SD], median [IQR]) were calculated for continuous variables, whereas frequency tables were compiled for categorical variables. We created an indicator variable for missing data for any of the included variables, and younger age was the only variable associated with any missing data. Multivariable logistic regression was used to assess whether veterans with PTSD who received couples or family-based psychotherapy treatment were more likely to complete a minimally adequate course of individual psychotherapy treatment. Factors in the model included type of couples or family-based treatment (if any was received during or after their individual trauma-focused treatment), and we included all measured patient characteristics as covariates. We conducted supplemental analyses in which we treated number of individual trauma treatment sessions as a continuous variable and used Poisson regression to model the count of individual psychotherapy sessions completed over 6 months (see eTable 4 in Supplement 1). Two-sided *P* < .05 was considered statistically significant. Analyses were conducted using SAS Enterprise Guide version 7.1 (SAS Institute) from May to August 2023.

## Results

### Demographic Characteristics, Military Service History, and Comorbid Conditions

The cohort consisted of 1 516 887 US veterans with VHA patient data who were newly diagnosed with PTSD between October 1, 2015, and December 31, 2019; 334 645 (23.5%) were Black, 1 006 168 (70.5%) were White, and 86 176 (6.0%) were other race; 1 322 592 (87.2%) were male; 1 201 902 (79.9%) lived in urban areas; and the mean (SD) age at first individual psychotherapy appointment was 52.7 (15.9) years (Table 1). With respect to comorbid conditions, 363 183 veterans (24.0%) were also diagnosed with depression, 496 065 (32.7%) with an anxiety disorder, 377 035 (24.9%) with a substance use disorder, and 162 306 (10.7%) with bipolar disorder, psychosis, or schizophrenia. We identified 239 238 veterans (21.6%) with a history of combat exposure, 152 037 (10.2%) had a history of military sexual trauma, and 1 220 652 (80.5%) had a service-connected disability rating greater than or equal to 50%.

**Table 1.** Descriptive Statistics of Study Participants

Variable	Participants, No. (%)	
	Whole cohort (N = 1 516 887)	Received family therapy subcohort (n = 58 653)
Gender		
Male	1 322 592 (87.2)	50 907 (86.8)
Female	194 295 (12.8)	7746 (13.2)
Race		
Black	334 645 (23.5)	10 878 (19.5)
White	1 006 168 (70.5)	41 572 (74.6)
Other^a^	86 176 (6)	3291(5.9)
Age, mean (SD), y	53 (15.9)	49 (15.4)
Living area type		
Rural	134 172 (8.9)	4891 (8.4)
Suburban	167 649 (11.2)	6301 (10.8)
Urban	1 201 902 (79.9)	47 066 (80.8)
Depression		
No	1 153 705 (76.1)	37 719 (64.3)
Yes	363 183 (23.9)	20 934 (35.7)
Anxiety		
No	1 020 823 (67.3)	32 012 (54.6)
Yes	496 065 (32.7)	26 641 (45.4)
Substance use		
No	1 139 853 (75.1)	38 357 (65.4)
Yes	377 035 (24.9)	20 296 (34.6)
Other mental health disorder		
No	1 354 582 (89.3)	48 512 (82.7)
Yes	162 306 (10.7)	10 141 (17.3)
Service-connected disability		
0%-40%	296 236 (19.5)	7281(12.4)
50%-100%	1 220 652 (80.5)	51 372 (87.6)
History of military sexual trauma		
No	1 339 901 (89.8)	51 213 (88.4)
Yes	152 037 (10.2)	6722 (11.6)
Combat exposure		
No	868 417 (78.4)	33 797 (78.3)
Yes	239 238 (21.6)	9380(21.7)
Family therapy type received		
None	1 458 235 (96.1)	NA
IBCT only	5210 (0.3)	5210 (8.9)
CBCT only	15 528 (1)	15 528 (26.5)
BFT only	282 (0.02)	282 (0.48)
Multiple types of family therapy	720 (0.05)	720 (1.2)
Family therapy undefined	36 913 (2.4)	36 913 (62.9)
Minimally adequate does of PE or CPT (≥8 sessions)		
No	1 352 431 (89.2)	48 092 (81.9)
Yes	164 457 (10.8)	10 561 (18)
At least 1 non-evidence-based practice mental health therapy session received		
No	660 500 (43.5)	6120 (10.4)
Yes	856 388 (56.5)	52 533 (89.6)

Abbreviations: BFT, behavioral family therapy; CBCT, cognitive-behavioral conjoint therapy; CPT, cognitive processing therapy; IBCT, integrative behavioral couples therapy; PE, prolonged exposure therapy; NA, not applicable.

^a^ Other race category included American Indian or Alaska Native (n = 21 104), Asian (n = 20 279), Pacific Islander (n = 19 094), and other/unknown (n = 25 699).

Among all cohort patients, 58 653 veterans (3.9%) received any family therapy; of these, 50 907 (86.8%) were male, 41 572 (74.6%) were White, 47 066 (80.8%) lived in urban locations; and 37 719 (64.3%) were not coping with depression (64.3%), 32 012 (54.6%) were not coping with anxiety, and 38 357 (65.4%) were not coping with substance use, and 48 512 (82.7%) were not coping with some other mental health disorder; 51 213 (88.4%) were not identified as having experienced military sexual trauma and 33 797 (78.3%) were not exposed to combat. Furthermore, 51 372 (87.6%) received more than or equal to 50% in total disability rating, and 52 533 (89.6%) had attended at least 1 other non-EBP treatment.

Within this subcohort of veterans who received any family therapy, there were 5210 (8.9%) who received IBCT therapy only, 15 528 (26.5%) who received CBCT therapy only, 282 (0.5%) who received BFT therapy only, 720 veterans (1.2%) who received more than 1 type of family therapy. There were 36 913 patients (62.9%) who received couples or family-based treatment based on *Current Procedural Terminology* codes, but the specific type of therapy was not indicated; and 10 561 veterans (18.0%) completed a minimally adequate amount of individual psychotherapy for PTSD.

### Statistical Analysis Results

Logistic regression was used to estimate a minimally adequate treatment of individual psychotherapy for PTSD among the 1 018 843 veterans who attended at least 1 treatment session (Table 2). Compared with receiving no family therapy care, veterans were more likely to complete a minimally adequate dose of individual PTSD treatment if they were also receiving CBCT (7% more likely; OR, 1.07 [95% CI, 1.01-1.13]), or undefined family therapy (68% more likely; OR, 1.68 [95% CI, 1.63-1.74]). However, veterans were 26% less likely (OR, 0.74 [95% CI, 0.66-0.82]) to complete a minimally adequate dose of individual PTSD treatment if they were also receiving IBCT compared with veterans receiving no family therapy care.

**Table 2.** Multivariable Logistic Regression Analysis Estimating Completion of a Minimally Adequate Dose of Individual PTSD Psychotherapy

Variable	β (95% CI)	OR (95% CI)	*P* value
Intercept	−3.37 (NA)	NA	<.001
Family therapy type (vs no family therapy)			
IBCT only	−0.31 (−0.42 to −0.20)	0.74 (0.66 to 0.82)	<.001
CBCT only	0.07 (0.01 to 1.12)	1.07 (1.01 to 1.13)	.03
BFT only	0.33 (−0.01 to 0.67)	1.39 (0.99 to 1.96)	.06
Multiple types	0.02 (−0.24 to 0.28)	1.02 (0.79 to 1.32)	.89
Undefined	0.52 (0.49 to 0.55)	1.68 (1.63 to 1.74)	<.001
Race (vs White)			
Black	−0.02 (−0.04 to −0.01)	0.98 (0.96 to 0.99)	.002
Other^a^	−0.12 (−0.15 to −0.09)	0.89 (0.86 to 0.91)	<.001
Gender (vs male)			
Female	0.10 (0.08 to 0.12)	1.10 (1.08 to 1.13)	<.001
Age at first therapy appointment	0.01 (0.01 to 0.01)	1.01 (1.01 to 1.01)	<.001
Living area type (vs rural)			
Suburban	−0.03 (−0.05 to 0)	0.97 (0.95 to 1.00)	.047
Urban	−0.10 (−0.13 to −0.08)	0.90 (0.88 to 0.92)	<.001
Depression (vs no)	0.33 (0.32 to 0.35)	1.40 (1.38 to 1.42)	<.001
Anxiety (vs no)	0.21 (0.19 to 0.22)	1.23 (1.21 to 1.25)	<.001
Substance use disorder (vs no)	0.62 (0.61 to 0.64)	1.87 (1.84 to 1.89)	<.001
Other mental health disorder (vs no)	0.64 (0.63 to 0.66)	1.90 (1.87 to 1.93)	<.001
Service-connected disability 50%-100% (vs 0%-40%)	0.01 (−0.01 to 0.03)	1.01 (0.99 to 1.03)	.28
History of military sexual trauma (vs no)	0.39 (0.37 to 0.42)	1.48 (1.45 to 1.52)	<.001
History of combat exposure (vs no)	−0.18 (−0.20 to −0.17)	0.84 (0.82 to 0.85)	<.001
At least 1 non–evidence-based practice mental health therapy session received (vs no)	0.48 (0.46 to 0.49)	1.61 (1.59 to 1.63)	<.001

Abbreviations: BFT, behavioral family therapy; CBCT, cognitive-behavioral conjoint therapy; IBCT, integrative behavioral couples therapy; NA, not applicable.

^a^ Other race category included American Indian or Alaska Native (n = 21 104), Asian (n = 20 279), Pacific Islander (n = 19 094), and other/unknown (n = 25 699).

In terms of demographic factors, veterans who were Black (OR, 0.98 [95% CI, 0.96-0.99]) or other minoritized race (OR, 0.89 [95% CI, 0.86-0.91) were slightly less likely to complete a minimally adequate dose of individual therapy compared with White veterans; and compared with male veterans, female veterans were more likely to complete a minimally adequate dose of individual therapy (OR, 1.10 [95% CI, 1.08-1.13]). For each 1-year increase in age, veterans were slightly more likely to complete a minimally adequate dose of treatment (OR, 1.01 [95% CI, 1.01-1.01]). Veterans living in urban areas were less likely (OR, 0.90 [95% CI, 0.88-0.92]), and veterans living in suburban areas were slightly less likely (OR, 0.98 [95% CI, 0.95-1.00) compared with patients living in rural areas. Veterans were more likely to complete a minimally adequate dose of individual trauma-focused therapy if they were diagnosed with depression (OR, 1.42 [95% CI, 1.40-1.43]), anxiety disorders (OR, 1.22 [95% CI, 1.20-1.24]), substance use disorders (OR, 1.87 [95% CI, 1.84-1.89]), or with bipolar disorder, psychosis, or schizophrenia (OR, 1.92 [95% CI, 1.89-1.95]) compared with veterans without these diagnoses.

Veterans with a history of military sexual trauma had 52% greater odds to complete a minimally adequate dose of individual trauma-focused therapy (OR, 1.52 [95% CI, 1.49-1.56]), and veterans with a history of combat exposure had 16% reduced odds to complete (OR, 0.84 [95% OR, CI, 0.83-0.85]) compared with those who did not have these histories. Lastly, veterans engaged in at least 1 non-EBP were more likely to have a minimally adequate dose of individual trauma-focused treatment (OR, 1.61 [95% CI, 1.59-1.63]).

## Discussion

In a cohort of more than 1.5 million VHA patients with PTSD, we found only approximately 4% had participated in any kind of couples or family therapy. As of 2020, about half of all military personnel were married, and about a third had at least 1 child, indicating a majority of veterans may have a close family member who could be included in treatment.^22^ Furthermore, prior work suggests that veterans have a preference for including their family in treatment for PTSD^23,24,25^ and VHA clinicians also have positive views regarding the importance of family-level treatment adjunctive to individual treatment for PTSD.^26^ Recent VHA data on the utilization of family therapy in mental health clinics shows that VHA patients diagnosed with PTSD report receiving more of any type of family therapy compared with those diagnosed with other mental health conditions.^27^ Additionally, there is strong evidence from systematic reviews documenting the effectiveness of couples therapies for treating adult PTSD and improving interpersonal relationship problems secondary to PTSD symptoms.^11,12,28^ Given the research linking PTSD and relational discord in veteran populations, this finding suggests potential underutilization of trauma-informed family therapy among VHA patients.

Although the specific therapy could not be identified for 63% of those who received couples/family treatment, CBCT was used most frequently among identifiable interventions (27%). Since there is not a standardized note template within the VHA to formally track clinicians using CBCT, there is a chance that some of the notes in the undefined family therapy category are CBCT. Veterans who participated in CBCT were more likely to complete a minimally adequate course of individual trauma-focused treatment. CBCT is the only family-based intervention offered in the VHA that has a PTSD focus, so it is unsurprising that it would be used the most in this cohort of veterans diagnosed with PTSD. This treatment directly involves the partner into the PTSD treatment in 3 phases over 15 sessions: (1) psychoeducation about PTSD and relationship safety, (2) communication skills and decreasing avoidance and emotional numbing, and (3) challenging trauma-related cognitions termed “stuck points.”^29^ These findings support the use of CBCT to help veterans in completing a minimally adequate course of individual trauma-focused treatment. Furthermore, these findings suggest the VHA should make efforts to support clinicians in reporting on the specific family therapies they are using (eg, rolling out a standardized note template for CBCT) as well as provide more training, consultation, and support for the delivery of this therapy.

Unexpectedly, we found that veterans diagnosed with PTSD and who attended at least 1 session of IBCT during their course of individual trauma-focused treatment were less likely to complete individual trauma-focused treatment. IBCT is not a trauma-focused treatment, rather it focuses on the health of the relationship overall. This finding could indicate that when a veteran diagnosed with PTSD is undergoing individual trauma-focused treatment, the addition of a separate evidence-based treatment may interfere with the veteran’s ability to complete their individual trauma care. Alternatively, veterans may experience substantial improvement in their functioning when undergoing ICBT, such that they do not need as much individual trauma-focused treatment. Because relationship issues are a key motivator for PTSD treatment engagement, veterans may prioritize relationship-focused issues if they are not accounted for within PE or CPT. To answer these questions, additional work is needed that assesses PTSD symptoms and functioning for veterans undergoing concurrent family therapy and individual trauma-focused treatment.

The potential for CBCT to increase adequate engagement in PE or CPT is important because retention in outpatient PTSD treatment is low^3,30^ and treatment retention in an individual course of therapy for PTSD is much more likely to result in a significant reduction in PTSD symptoms.^31,32^ Our findings are consistent with preliminary research that suggests that brief family involvement (ie, 2 sessions of psychoeducation and skill building) in the veteran’s individual treatment of PTSD can improve treatment retention in individual therapy.^7^ Further research is needed to better understand the associations between family therapies and PTSD symptoms, functioning, and quality of life outcomes in the VHA. Additionally, future research should focus on the mechanisms behind how these therapies might improve engagement and comparative effectiveness studies between the different types of family treatments offered. Additional research could help determine if it is better when the family is involved in the PTSD treatment itself or if receiving therapy for relationship issues helps improve individual PTSD treatment retention.

### Limitations

This study had limitations. The use of EHR data provided access to a very large sample of VHA patients; however, due to the lack of standardized methods for recording the type of couples and family therapy provided, the automated search term strategy used was not able to identify the type of couple or family therapy for approximately 63% of patients who participated in family-level treatment. There is also a chance that veterans could have had a family session in their PE or CPT treatment that was not documented in *Current Procedural Terminology* codes we searched. Due to the observational nature of the study, we were unable to make causal inferences regarding the effect of couples or family therapy on PE or CPT completion. Though small-scale randomized clinical trial evidence supports trauma-informed family treatment as being effective for individual treatment completion,^2^ it is also possible that factors such as perceived need for treatment or treatment acceptability may explain why patients who seek out trauma-informed couples or family therapy are more likely to complete individual treatment. The current study did not account for severity of PTSD symptoms. A recent study of Operation Enduring Freedom and Operation Iraqi Freedom veterans found that more severe PTSD symptoms were associated with a greater likelihood of family-involved care.^23^ Perhaps veterans with less severe symptoms are less in need of family treatment because their symptoms are not as detrimental to the functioning of the relationship.

## Conclusions

This study’s findings from a very large cohort of VHA patients diagnosed with PTSD suggest that trauma-informed family-level therapies are possibly underutilized but may be helpful in terms of their potential to support adequate engagement in effective individual treatment for PTSD. These findings support growing calls to evaluate the effectiveness of and increased provision of family-based services to veterans with PTSD through the VHA,^23,33,34,35^ recommendations which were included in the Institute of Medicine’s seminal report on the treatment of PTSD among military and veteran populations more than a decade ago in 2012.^36^

## Notes

Supplement 1. **eTable 1.** Posttraumatic Stress Disorder International Classification of Diseases 10 Codes (ICD10) Used in Analyses **eTable 2.** List of Current Procedural Terminology (CPT) for Couples/Family-Based Psychotherapy With the Veterans Health Administration **eTable 3.** Health Factors Field and Note Content Search Terms for Evidence-Based Family Therapy Within the Veterans Health Administration **eTable 4.** Poisson Regression Output Estimating Individual PTSD Psychotherapy Session Count

Supplement 2.
Data Sharing Statement
