# Supplementary material for: Retention in Individual Trauma-Focused Treatment Following Family-Based Treatment Among US Veterans
Source: JAMA Netw Open. 2023 Dec 21;6(12):e2349098. doi: 10.1001/jamanetworkopen.2023.49098 (PMC10739069; doi:10.1001/jamanetworkopen.2023.49098)
Supplement: Supplement 1. — eTable 1. Posttraumatic Stress Disorder International Classification of Diseases 10 Codes (ICD10) Used in Analyses eTable 2. List of Current Procedural Terminology (CPT) for Couples/Family-Based Psychotherapy With the Veterans Health Administration eTable 3. Health Factors Field and Note Content Search Terms for Evidence-Based Family Therapy Within the Veterans Health Administration eTable 4. Poisson Regression Output Estimating Individual PTSD Psychotherapy Session Count [file jamanetwopen-e2349098-s001.pdf]

Supplemental Online Content

Dodge J, Sullivan K, Grau PP, Chen C, Sripada R, Pfeiffer PN. Retention in individual trauma-focused treatment following family-based treatment among US veterans. *JAMA Netw Open.* 2023;6(12):e2349098. doi:10.1001/jamanetworkopen.2023.49098

eTable 1. Posttraumatic Stress Disorder International Classification of Diseases 10 Codes (ICD10) Used in Analyses

eTable 2. List of Current Procedural Terminology (CPT) For Couples/Family-Based Psychotherapy With the Veterans Health Administration

eTable 3. Health Factors Field and Note Content Search Terms for Evidence-Based Family Therapy Within the Veterans Health Administration

eTable 4. Poisson Regression Output Estimating Individual PTSD Psychotherapy Session Count

This supplemental material has been provided by the authors to give readers additional information about their work.

© 2023 Dodge J et al. *JAMA Network Open.*

eTable 1: Posttraumatic Stress Disorder International Classification of Diseases 10 Codes (ICD10) Used in Analyses.

ICD10	Long Label for ICD10
F431	Post-traumatic stress disorder (PTSD)
F4310	Post-traumatic stress disorder, unspecified
F4311	Post-traumatic stress disorder, acute
F4312	Post-traumatic stress disorder, chronic

© 2023 Dodge J et al. *JAMA Network Open.*

eTable 2: List of Current Procedural Terminology (CPT) For Couples/Family-Based Psychotherapy with the Veterans Health Administration.

*Family/Couples Psychotherapy with the patient present
○ 90847
**PTSD-related mental health psychotherapy session
○ 90832
○ 90834
○ 90837
○ 90853

*While other CPT codes suggest family-based therapy, such as a family therapy session and the patient not present (90846), mental health education or communication with family members (90887), or multifamily group psychotherapy, we decided to take a conservative approach and focus on the CPT code that reflected when both Veteran and their respective family were present for an evidence-based treatment.

**These codes were used to create the variable assessing receipt of any non-EBP mental health visit

© 2023 Dodge J et al. *JAMA Network Open.*

eTable 3. Health Factors Field and Note Content Search Terms for Evidence-Based Family Therapy within the Veterans Health Administration.

**Type of Family Therapy**	***Health Factor Field or Note Content Search Terms**
Behavioral Family Therapy	Health Factor Field:<br>“BFT”<br>“Behavioral Family Therapy”
Integrative Behavioral Couples Therapy	Health Factor Field:<br>“IBCT”<br>“Integrative Behavioral Couples Therapy”<br>“Integrative Behavioral Couple Therapy”
Cognitive Behavioral Conjoint Therapy	Note Contents:<br>“CBCT”<br>“Cognitive Behavioral Conjoint Therapy”<br>Exclude:<br>“dentist”<br>“dental”<br>“radiation”

*****Only treatments that have a standardized note template have a Health Factors Field.

© 2023 Dodge J et al. *JAMA Network Open.*

eTable 4. Poisson Regression Output Estimating Individual PTSD Psychotherapy Session Count

Variable	Category	Degrees of Freedom	Estimate & Wald 95% CI	Standard Error	Wald Chi-Square	P-Val
Intercept	-	1	0.47 (0.46, 0.47)	0.00	18,328	<.0001
Family Therapy Type (vs. No Family Therapy)	IBCT Only	1	-0.14 (-0.16, -0.12)	0.01	221	<.0001
	CBCT Only	1	0.03 (0.02, 0.04)	0.01	31	<.0001
	BFT Only	1	0.20 (0.14, 0.26)	0.03	44	<.0001
	Multiple Types	1	0.02 (-0.23, 0.06)	0.02	0.55	0.46
	Undefined	1	0.32 (0.32, 0.33)	0.00	13,521	<.0001
Race (vs. White)	Black	1	-0.01 (-0.01, -0.00)	0.00	22	<.0001
	Other	1	-0.07 (-0.08, -0.07)	0.00	784	<.0001
Gender (vs. Male)	Female	1	0.05 (0.05, 0.06)	0.00	646	<.0001
Age at First Therapy Appointment	-	1	0.003 (0.003, 0.003)	0.00	5,421	<.0001
Living Area Type (vs. Rural)	Suburban	1	-0.02 (-0.03, -0.02)	0.00	84	<.0001
	Urban	1	-0.07 (-0.07, -0.06)	0.00	1,146	<.0001
Depression	Yes	1	0.22 (0.22, 0.22)	0.00	32,147	<.0001
Anxiety	Yes	1	0.16 (0.16, 0.17)	0.00	1,8260	<.0001
Substance Use Disorder	Yes	1	0.42 (0.42, 0.42)	0.00	116,830	<.0001
Other Mental Health Disorder	Yes	1	0.42 (0.42, 0.43)	0.00	84,852	<.0001
Service-Connected Disability (vs. 0-40%)	50-100%	1	0.02 (0.02, 0.02)	0.00	210	<.0001
History of Military Sexual Trauma	Yes	1	0.29 (0.28, 0.29)	0.00	20,883	<.0001
History of Combat Exposure	Yes	1	-0.11 (-0.12, -0.11)	0.00	5,663	<.0001
At Least One Non-	Yes	1	0.31 (0.31, 0.31)	0.00	56,597	<.0001

© 2023 Dodge J et al. *JAMA Network Open.*

Evidence-Based Practice Mental Health Therapy Session Received						

© 2023 Dodge J et al. *JAMA Network Open.*
