# Supplementary material for: Retention in Individual Trauma-Focused Treatment Following Family-Based Treatment Among US Veterans
Source: JAMA Netw Open. 2023 Dec 21;6(12):e2349098. doi: 10.1001/jamanetworkopen.2023.49098 (PMC10739069; doi:10.1001/jamanetworkopen.2023.49098)
Supplement: Supplement 2. — Data Sharing Statement [file jamanetwopen-e2349098-s002.pdf]

Data Sharing Statement

Dodge. Retention in Individual Trauma-Focused Treatment Following Family-Based Treatment Among US Veterans. *JAMA Netw Open*. Published December 21, 2023.
doi:10.1001/jamanetworkopen.2023.49098

Data
Data available: No

Additional Information
Explanation for why data not available: Data is not available for public access because institutional policies require that data use agreements have to be in place before data can be shared.
